# Long-Term Visual Acuity in Young Children After Lensectomy for Congenital Cataract

**DOI:** 10.3390/vision10040069

**Published:** 2026-09-12

**Authors:** Caroline Glänzer, Sami Dalbah, Jan Strathmann, Mareile Knetsch, Anja Eckstein, Nikolaos E. Bechrakis, Henrike Westekemper, Mael Lever

**Affiliations:** 1 Department of Ophthalmology, University Hospital Essen, 45147 Essen, Germany; 2 Artemis Eye Clinic Tausendfensterhaus, 47119 Duisburg, Germany

**Keywords:** lensectomy, aphakia, ocular dysgenesis, contact lens, persistent fetal vasculature, postoperative glaucoma, visual rehabilitation, amblyopia

## Abstract

(1) Background: Congenital cataract is a rare but severe childhood ocular disorder that can lead to permanent visual impairment if left untreated. Factors influencing long-term visual outcomes are insufficiently defined. The aim of this study was to evaluate long-term visual outcomes after congenital cataract surgery and to identify factors associated with visual acuity impairment and reoperation rates. (2) Methods: This retrospective cohort study included 173 eyes of 116 patients who underwent surgery for congenital cataract at the University Hospital Essen between 2000 and 2018. Patients with a minimum postoperative follow-up of six years were included. Best-corrected visual acuity (BCVA) was analyzed according to binocular condition, age at surgery and the presence of comorbidities. Postoperative complications and the need for additional surgical interventions were evaluated. (3) Results: Unilateral cataract was present in 48% (n = 56) and bilateral cataract in 52% (n = 60) of patients. Age at surgery ranged from 2.1 weeks to 15.6 years in unilateral cases and from 3.1 weeks to 8.7 years in bilateral cases. Six years after surgery, mean BCVA was 1.0 logMAR (Snellen 20/200) in unilateral cases and 0.4 logMAR (Snellen 20/50) in bilateral cases. No significant association was found between long-term BCVA and either age at surgery or ocular or systemic comorbidities. Patients with microphthalmia and those born prematurely showed significantly higher rates of additional surgical procedures. (4) Conclusions: Bilateral cases of congenital cataract achieved a significantly better visual acuity than unilateral cases after surgery. In this cohort, neither age at surgery nor the presence of comorbidities was significantly associated with visual acuity, whereas microphthalmia and prematurity were linked to an increased likelihood of additional surgical interventions.

## 1. Introduction

Congenital cataract is a rare but serious pediatric ocular disorder, with a worldwide incidence of 4.2 per 10,000 newborns [1]. Without adequate treatment, it can lead to significant visual impairment, adversely affecting long-term quality of life. Early diagnosis and timely surgical intervention are essential to prevent irreversible amblyopia [2,3].

Advances in early diagnosis, surgical techniques and postoperative care have improved visual outcomes over the past decades [4,5]. Standardized procedures for early detection and lens extraction, combined with posterior capsulotomy and anterior vitrectomy, have been established to promote optimized visual development and to reduce complications during follow-up. Furthermore, improvements in preoperative diagnostics and both primary and secondary intraocular lens (IOL) implantation have contributed to better planning and execution of surgery.

The visual prognosis largely depends on the prevention of refractive amblyopia. In cases where primary IOL implantation is not performed, aphakia is corrected by fitting contact lenses or aphakic glasses. A second essential component of amblyopia prevention or therapy is occlusion therapy, most often part-time occlusion, where the functionally superior eye is temporarily covered. The goal of occlusion therapy is to promote the maturation of the visual cortex in early childhood through targeted stimulation of the amblyopic eye [6]. Particularly in unilateral cataract, intensive occlusion therapy immediately after surgery is crucial for achieving the best possible visual outcome in the affected eye. The success of both contact lens fitting and occlusion therapy largely depends on the child’s cooperation and parental adherence, which have been shown to be important determinants of visual outcome following surgery [7,8].

Despite these advances, long-term outcomes following surgical treatment remain insufficiently studied, especially regarding the impact of associated factors. These include age at surgery, the type of refractive correction, comorbidities, the need for reoperations, and postoperative complications such as glaucoma.

Therefore, the aim of the present study was to evaluate the long-term functional outcome after surgical treatment of congenital cataract and to identify influencing factors. Attention was paid to the effects of uni- or bilaterality, age at surgery and postoperative refractive correction on visual acuity and the role of comorbidities or the need for revision surgery.

## 2. Materials and Methods

### 2.1. Study Design

This retrospective study included patients aged between 1.7 weeks to 15.6 years with unilateral or bilateral congenital cataract who underwent lensectomy and had a minimum postoperative follow-up of six years. Exclusion criteria were acquired cataracts (e.g., due to trauma or inflammation), lensectomy performed for other reasons (e.g., lenticonus or lens subluxation), and incomplete postoperative follow-up data. In total, 301 eyes of 248 patients were excluded.

This study was conducted in accordance with the Declaration of Helsinki and was approved by the ethics committee of the University Hospital Essen, Germany (24-12089-BO).

### 2.2. Data Collection

Data were extracted from medical records of the Ophthalmology Department. Variables included uni- or bilaterality, age at surgery, method of postoperative refractive correction (contact lenses, spectacles, IOL implantation), the need for reoperations and the presence of comorbidities. To assess visual outcomes, best-corrected visual acuity (BCVA) was recorded at one, two, four, and six years postoperatively. All visual acuity values included were converted to logarithm of the minimum angle of resolution (logMAR) before statistical analysis. Visual acuity testing was performed using age-appropriate standardized charts (Cardiff test, LEA symbols, tumbling E, Snellen numbers or Landolt rings).

### 2.3. Surgical Procedure

Prior to congenital cataract surgery, examination under general anesthesia was performed to allow detailed assessment of the lens and other ocular structures. This included measurement of intraocular pressure, biometry (total length, keratometry), corneal radius, corneal thickness, and fundoscopy to optimize preoperative planning.

All procedures were performed under general anesthesia. Surgical access was obtained via two 1.0 mm clear cornea incisions at the temporal and nasal positions. Trypan blue (VisionBlue^®^, D.O.R.C. Dutch Ophthalmic Research Center, Zuidland, The Netherlands) was instilled to stain the anterior capsule, followed by injection of viscoelastic. An anterior capsulorhexis was performed using Hattenbach forceps, and lens material was aspirated using an irrigation/aspiration system. Then, a posterior capsulotomy and anterior vitrectomy were carried out.

For IOL implantation, prior to posterior capsulorhexis, the anterior chamber was refilled with viscoelastic. A sclero-corneal tunnel incision at the 12 o’clock position was created to implant the intraocular lens into the capsular bag. Both, a posterior capsulotomy and anterior vitrectomy were performed and the viscoelastic was removed. All incisions were closed with 10-0 nylon sutures. Finally, 1.0 mg subconjunctival dexamethasone was administered. Sutures were removed 1 week after surgery in inhalation anesthesia. The general surgical approach and postoperative management remained largely consistent throughout the study period.

### 2.4. Postoperative Care and Follow-Up

In the first days after surgery, patients were monitored daily to monitor the eye pressure and intraocular inflammation, and detect complications at the earliest stage. Follow-up examinations included assessment of age-appropriate visual acuity, measurement of intraocular pressure for early detection of glaucoma, evaluation of the capsular bag for posterior capsular opacification, and retinoscopy for refractive correction. Long-term follow-up was performed at intervals of approximately three to six months to monitor visual development and detect late complications. For amblyopia treatment, occlusion therapy was recommended as follows: In children with unilateral cataract, daily patching of the fellow eye was recommended for approximately half of the child’s waking hours (up to 6 h). In bilateral cases, daily occlusion of the better-seeing eye was recommended for one hour for each year of life only in cases where a difference of visual acuity test was noticed. Postoperative glaucoma was diagnosed primarily based on elevated intraocular pressure. In young children, additional clinical signs such as buphthalmos, increasing axial lengths and corneal changes were also considered. Glaucoma was managed medically or surgically depending on the underlying cause and clinical severity.

### 2.5. Statistical Analysis

Data were collected using Microsoft Excel (Microsoft, Redmond, WA, USA, version 2605) and analyzed with GraphPad Prism (version 11.0.2; GraphPad Software, LLC, Boston, MA, USA). Differences between two groups were evaluated using the t-test or Mann–Whitney U test. For comparisons across more than two groups, the Kruskal–Wallis test was applied, followed by Dunn’s multiple comparisons test to adjust for multiple testing. Categorical variables were tested for statistical significance using Fisher’s exact test. Univariable and multivariable logistic regression analyses were performed to evaluate the association between age at surgery and visual outcome as well as the association between clinical variables and visual outcome in the overall cohort. For this analysis, visual acuity was dichotomized according to the definition of the World Health Organization for “low vision” (visual acuity > 0.5 logMAR or Snellen 20/70 and worse). In patients with bilateral cataract, the better- and worse-seeing eyes were analyzed separately, and both eyes of the same patient were not included as independent observations within the same statistical analysis. *p*-values < 0.05 were considered statistically significant.

## 3. Results

### 3.1. Patient Characteristics

This retrospective study analyzed data from 173 eyes of 116 patients (51% males) who underwent surgery for congenital cataract at the University Hospital Essen between 2000 and 2018. Unilateral cataract was present in 48% (n = 56) of patients, while 52% (n = 60) were affected bilaterally. The median age at surgery was 4.9 months (IQR 3.4–17.3 months, range: 0.5–187.2 months) in unilateral cases and 3.4 months (IQR 2.0–5.4 months, range: 0.7–104.0 months) in bilateral cases.

Patients were categorized into three subgroups according to age at surgery: 52% (n = 90) underwent surgery up to the age of 4 months, 31% (n = 53) between 5 and 11 months, and 17% (n = 30) at 12 months or later. Patients with bilateral cataract underwent surgery significantly earlier than patients with unilateral cataract (mean age at surgery: 9.1 vs. 14.9 months, *p* = 0.0008). Accordingly, surgery within the first 4 months of life was performed significantly more often in bilateral than in unilateral cases (58% vs. 39%), whereas surgery after 12 months of age was more common in unilateral than in bilateral cases (29% vs. 12%).

Postoperative refractive correction was more frequently achieved with contact lenses (68%, n = 117), whereas 13% (n = 22) received aphakic spectacles and 12% (n = 20) underwent primary intraocular lens implantation. Intraocular lens implantation was performed significantly more often in unilateral than in bilateral cases (20% vs. 8%) (Table 1).

### 3.2. Impact of Unilaterality of Cataract on Visual Acuity

Long-term visual outcome differed significantly between unilateral (n = 56) and bilateral congenital cataract cases (n = 117). Six years after surgery, the mean visual acuity in the unilateral group was 1.0 logMAR (Snellen 20/200), whereas patients with bilateral cataract achieved a mean visual acuity of 0.4 logMAR (Snellen 20/50) on the better-seeing eye and 0.5 logMAR (Snellen 20/63) on the worse-seeing eye. Both the differences between unilateral eyes and the better-seeing bilateral eyes or the worse-seeing bilateral eyes were statistically significant (Table 2).

### 3.3. Impact of Age at Surgery on Visual Acuity

**Figure 1:**
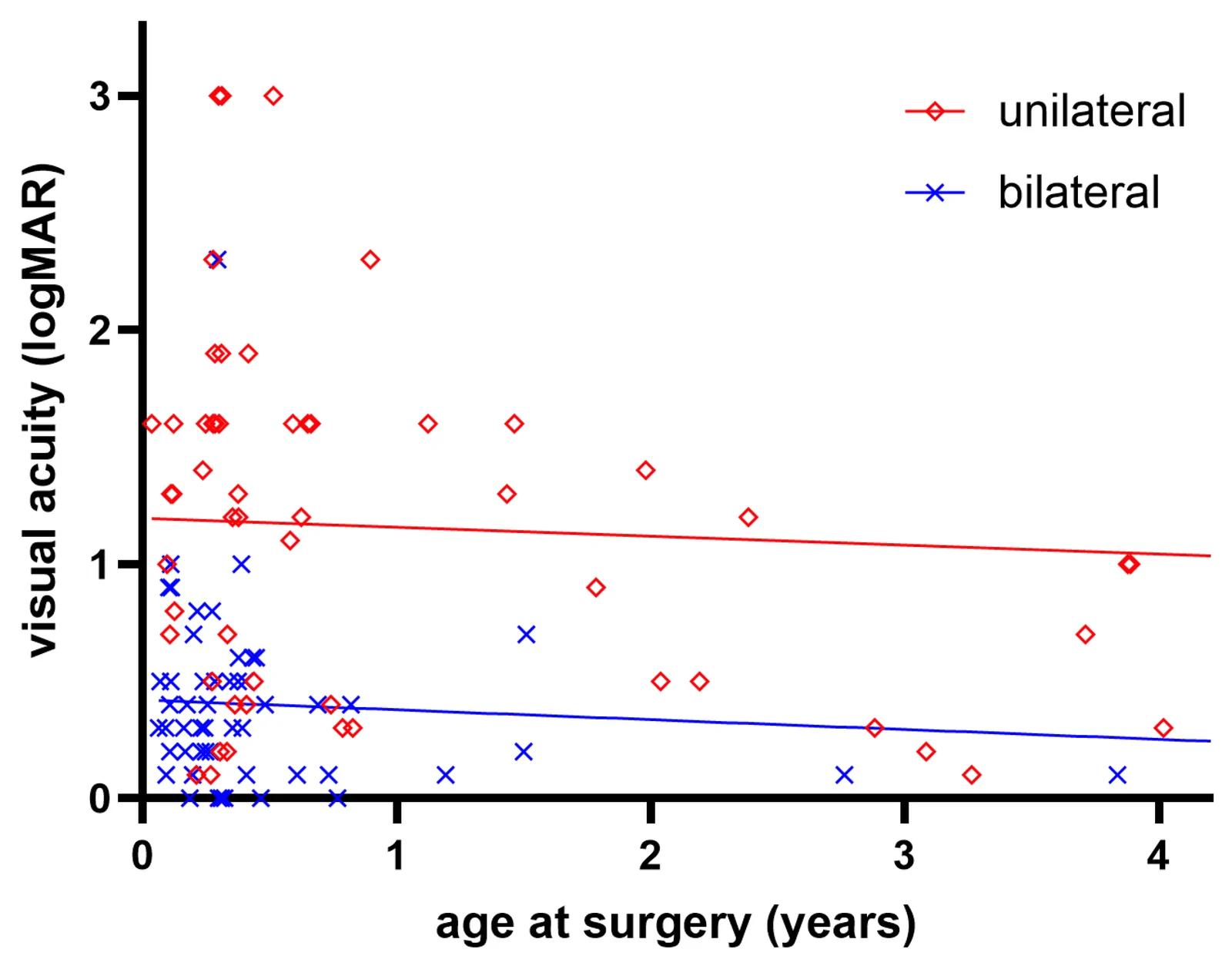
Association between age at surgery and long-term visual acuity in unilateral and bilateral congenital cataract. Unilateral cases are shown in red, bilateral cases in blue. Visual acuity is shown in logMAR.

To assess the influence of age at surgery on the long-term visual outcome, patients were divided into three subgroups (age at surgery ≤ 4 months, 5–11 months, and ≥12 months). These age groups reflect, on one hand, the therapeutic goal of treating cataracts present at birth in the first months of life and, on the other hand, developmental milestones like the onset of independent walking and increasing interest in distant objects typically reached around 12 months of age. Furthermore, initial contact lens fitting and handling become more challenging with children aged one year and older. Overall, VA was best in patients who underwent surgery after their 1st birthday and poorest in those who were up to 4 months old at the time of surgery. In unilateral patients, the best mean visual acuity was observed in those operated on after 12 months (0.9 logMAR, Snellen 20/160), while the poorest outcomes occurred in patients operated on before 4 months of age (1.3 logMAR, Snellen 20/400) (Table 3). In bilateral cases, mean visual acuity of the better eye ranged from 0.2 logMAR (Snellen 20/32) to 0.4 logMAR (Snellen 20/50), without statistically significant differences between subgroups (Table 3). Figure 1 visualizes the relationship between age at surgery and visual acuity in unilateral and bilateral cases. Notably, in bilateral cataract cases, visual acuity of at least 1.0 logMAR was achieved in nearly all patients across the different age groups. In contrast, unilateral cases showed substantially poorer outcomes, with a considerable proportion of eyes remaining below this threshold.

### 3.4. Impact of Postoperative Refractive Correction on Visual Acuity

To assess the impact of the postoperative refractive correction method on the long-term visual outcome, visual acuity six years after surgery was analyzed comparing patients who were corrected with contact lenses or spectacles, or were operated on with primary implantation of an IOL. Contact lens correction was used in 84% of unilateral cases and 73% of bilateral cases. Of the 20 eyes that underwent primary IOL implantation, 11 were unilateral and 9 were bilateral cases. No significant differences in long-term visual acuity were found between the different forms of postoperative refractive correction (Table 4). The mean age of patients that underwent primary IOL implantation was 53 months and was significantly higher than in patients treated with contact lenses or spectacles. Therefore, contact lens and spectacle correction were additionally compared separately using the Mann–Whitney U test. This analysis revealed no significant differences regarding either age at surgery (*p* = 0.28) or long-term visual acuity after six years (*p* = 0.39).

### 3.5. Impact of Comorbidities on Visual Acuity

When comparing patients with and without comorbidities (microphthalmia, persistent fetal vasculature (PFV), prematurity, developmental retardation, or syndromic diseases), a trend towards a better visual acuity in eyes without comorbidity was present, but without reaching statistical significance. Among unilateral patients, the mean visual acuity was 1.1 logMAR (Snellen 20/250) in those with comorbidities versus 0.9 logMAR (Snellen 20/160) in those without. In the bilateral group, the better eye achieved 0.4 logMAR (Snellen 20/50) in patients with comorbidities and 0.3 logMAR (Snellen 20/40) in those without (Table 5).

Additional analyses were performed to differentiate between ocular (microphthalmia and PFV) and systemic comorbidities (prematurity, developmental retardation and syndromic diseases). Ocular comorbidities were significantly more common in unilateral than in bilateral cases (*p* = 0.001), whereas systemic comorbidities were shown more often in bilateral cases, although this difference was not statistically significant (*p* = 0.08). Furthermore, the impact of ocular and systemic comorbidities on visual outcome was analyzed separately. Ocular comorbidities were not significantly associated with visual acuity after six years in neither unilateral (*p* = 0.14) nor bilateral cases (better eye) (*p* = 0.38). Likewise, no significant association was found for systemic comorbidities in unilateral (*p* = 0.96) or bilateral cases (better eye) (*p* = 0.36).

### 3.6. Impact of Comorbidities on Reoperation Rates

To further evaluate postoperative outcomes, the occurrence of complications and additional surgical interventions during follow-up was analyzed. Surgical secondary capsulotomy was necessary in 10% (n = 18) of cases, and another 10% (n = 17) underwent anterior chamber revision. Additionally, 6% (n = 12) were operated on for strabismus during follow-up.

Overall, 24% (n = 41) of all eyes developed glaucoma, of which 56% (n = 21) required surgical intervention. Among the 26 eyes operated on before the sixth week of life, glaucoma occurred particularly frequently: in 25% (n = 1) of unilateral and 41% (n = 9) of bilateral cases. Among the eyes with surgical treatment for postoperative glaucoma, the median interval between cataract surgery and glaucoma surgery was 64.5 months (IQR 46.3–121.3 months).

Evaluation of the association between comorbidities and reoperations (anterior chamber revision or secondary capsulotomy) showed that children with microphthalmia and those born prematurely had a significantly higher rate of reoperations. Other comorbidities did not show a significant correlation with the need for additional surgery (Table 6).

### 3.7. Logistic Regression Analysis of Factors Associated with Visual Outcome

To evaluate factors associated with long-term visual outcome, visual acuity was dichotomized at 0.5 logMAR, corresponding to the World Health Organization’s definition of “low vision”, and univariable logistic regression analysis was performed. Among the variables tested, unilaterality was identified as the only factor strongly associated with visual outcome. Unilateral manifestation was associated with higher odds of achieving a VA > 0.5 logMAR (Snellen 20/70 Snellen or worse) compared to bilateral cases. Also, the presence of comorbidities showed higher odds for the development of low vision. However, for age at surgery, analyzed as a continuous variable and as age groups, and for the need for revision surgery, the precision of the estimates—as measured by the width of the 95% CI—was so low that a substantive interpretation was not reasonable. In our multivariable logistic regression model, however, only unilaterality was statistically associated with low vision (Table 7).

## 4. Discussion

In the present study, we investigated the long-term visual outcome after surgical treatment of congenital cataract. We found that patients with bilateral cataract achieved significantly better visual acuity six years postoperatively compared to those with a unilateral cataract. In contrast, the impact of age at surgery on long-term visual outcome could not be clearly demonstrated, and the presence of additional comorbidities was not significantly associated with a worse visual outcome. However, patients with microphthalmia and prematurity showed a significantly higher rate of reoperations.

Regarding bilaterality, we observed better visual acuity in bilateral than in unilateral cases six years after surgery. Furthermore, univariable and multivariable regression analysis identified unilateral manifestations as the factor most strongly associated with poorer visual outcome: in our cohort unilateral cataract was associated with more than five to six times higher odds of low vision compared to bilateral cases. However, unilateral patients in our cohort were operated on at a significantly older age than bilateral patients, which may have contributed to the poorer visual outcome in addition to bilaterality itself. The British Congenital Cataract Study by Chak et al. demonstrated that children with bilateral cataract achieved better median visual acuity than those with unilateral disease, with 50% of bilateral cases but only 20% of unilateral cases achieving at least 0.5 logMAR (Snellen 20/60) [2]. The Toddler Aphakia and Pseudophakia Studies (TAPS) confirmed this difference in children operated on between 7 and 24 months of age, reporting severe visual impairment in 44% of unilateral cases, whereas 94% of the better-seeing eyes in bilateral cases achieved at least 0.6 logMAR (Snellen 20/80) [9,10]. More recently, Repka et al. also identified bilaterality as an important determinant for postoperative visual acuity after congenital cataract surgery when performed within the first 6 months of life [11]. Our findings are consistent with the known high risk for severe deprivation amblyopia in unilateral congenital cataract, where interocular competition leads to poorer visual development, whereas bilateral involvement allows for more balanced visual maturation.

We then looked at the effect of age at lensectomy on the long-term visual acuity. No significant association between age at surgery and visual outcome was found in unilateral or bilateral cases, neither when predefined age groups were compared nor when age was analyzed as a continuous variable using logistic regression. However, surgical timing may still influence visual outcome despite the lack of a statistical association in our study. In the existing literature, the impact of timing of surgery is evaluated inconsistently. Lambert described an optimal surgical window between the 4th and 6th week of life in the management of congenital cataract [12]. Similarly, Bremond-Gignac et al. recommended surgery at approximately 6 weeks of age for unilateral cataracts and before 8 weeks for bilateral cases to prevent deprivation amblyopia [13]. More recent reviews have supported these recommendations. While emphasizing the need to balance the risk of deprivation amblyopia due to delayed treatment against the increased risk of complications, particularly glaucoma, associated with early surgery, Lenhart et al. also recommended surgery after 4 weeks of life, whereas Lloyd et al. proposed optimal surgical windows of 6 to 8 weeks for unilateral and 6 to 10 weeks for bilateral congenital cataracts [14,15]. In contrast, the randomized controlled trial by Lin et al. reported significantly better visual outcomes in bilateral cataract patients operated on at 6 months of age compared to those operated on at 3 months [16]. These divergent findings indicate that the observed relationship between surgical timing and visual outcome may be influenced by clinical characteristics, including the severity and extent of lens opacity, ocular comorbidities and factors related to postoperative visual rehabilitation. In particular, children with dense, visually significant cataracts may undergo surgery at an earlier age than children with less severe lens opacities. Differences in cataract severity may therefore have contributed to the descriptively poorer visual outcomes observed in some of the patients who were operated on earlier in our cohort. However, a later age at surgery does not necessarily mean that a severe, visually significant cataract has been present since birth. In some children, the lens opacity may initially have been less pronounced and thus was not detected until a progression occurred. Our cohort included a broad spectrum of congenital cataracts requiring surgical treatment. However, cataract morphology and severity were not documented in a standardized manner to allow a reliable classification according to the degree of preoperative visual deprivation. Therefore, the influence of cataract severity on long-term visual outcome and on other parameters analyzed in this study could not be assessed reliably.

The use of predefined age categories and unequal group sizes may have limited the statistical power to detect an association between age at surgery and visual outcome. In our cohort, patients with unilateral cataracts were operated on at a significantly older age than patients with bilateral cataracts. One possible explanation for this finding could be delayed diagnosis of unilateral cataracts: as our study includes patients operated on between 2000 and 2018, some children were diagnosed before the introduction of mandatory red reflex test (Brückner test) in pediatric examinations. This may have affected the detection of unilateral cases, in which visual impairment is often less noticeable for parents. Our findings therefore highlight the importance of early screening programs and close collaboration between pediatrics and ophthalmologists, as early diagnosis remains an important determinant for optimal visual rehabilitation [2].

In our cohort, no significant influence of the type of postoperative refractive correction on long-term visual outcome could be demonstrated. However, differences in age at surgery need to be considered when comparing these treatment groups, as patients undergoing primary IOL implantation were substantially older than patients receiving contact lenses or spectacle correction. Consequently, the similar visual outcomes observed in our cohort do not necessarily indicate comparable effectiveness of the different forms of refractive correction treatment. Similar findings were reported by Lambert et al. in the long-term follow-up of the Infant Aphakia Treatment Study (IATS), which compared visual outcomes after unilateral congenital cataract surgery in children treated with either primary IOL implantation or contact lenses [17]. After 10.5 years of follow-up, no significant difference in visual acuity was found between the two groups. The authors therefore concluded that primary IOL implantation does not necessarily lead to a better visual outcome and that both approaches can achieve comparable visual rehabilitation. Likewise, Sengör et al. concluded in their review that contact lens correction represents an effective method of visual rehabilitation after congenital cataract surgery and can achieve visual outcomes comparable to other forms of refractive correction when early surgery, consistent wear and adherence to amblyopia treatment are maintained [18].

Additionally, in our cohort, the presence of additional comorbidities was not significantly associated with worse long-term visual acuity in either unilateral or bilateral cases. In contrast, Bradford et al. identified structural ocular anomalies as well as systemic comorbidities as predictors of poorer long-term visual outcomes [19]. Likewise, the British Congenital Cataract Study by Chak et al. demonstrated that, among children undergoing bilateral cataract surgery, the presence of non-ophthalmic comorbidities was significantly associated with an increased likelihood of worse visual acuity, whereas no such association could be demonstrated in unilateral cases [2]. Although subgroup analysis did not reveal statistically significant differences, univariable logistic regression showed a weak association between the presence of comorbidities and the risk of low vision. Our findings therefore suggest that satisfactory visual outcomes can be achieved even in the presence of comorbidities, provided that structured long-term follow-up, including regular assessment of visual acuity, refractive development, and intraocular pressure is implemented.

In contrast, our data revealed a clear association between certain comorbidities and the need for reoperations. Patients with microphthalmia and those born prematurely had a significantly higher rate of additional surgical procedures. In contrast, Morrison et al., in a subanalysis of the Infant Aphakia Treatment Study (IATS), reported that the presence of PFV was not associated with an increased rate of additional intraocular procedures during the first postoperative year [20]. However, Warren et al. demonstrated that more severe forms of PFV are associated with a higher rate of postoperative complications and additional surgical interventions [21]. Taken together, these findings suggest that not all comorbidities affect the postoperative course to the same extent. While PFV alone may not increase the need for additional surgery, structural ocular abnormalities such as microphthalmia appear to represent important risk factors for postoperative interventions.

In addition to what was previously discussed, further limitations need to be considered when interpreting our results. First, due to the retrospective study design, data collection was not fully standardized. Furthermore, due to the wide range of age and different cognitive abilities of the children, different age-appropriate visual acuity tests had to be used throughout follow-up, which may have affected the comparability of visual acuity measurements and increased variability in the documented outcomes. Subgroup sizes were partly unequal, which may have affected group comparisons. Another important limitation is the lack of reliable data on amblyopia therapy. Although a general occlusion protocol was recommended, treatment was individually adapted according to clinical findings and visual development, and actual occlusion times were not reliably documented throughout the follow-up period: when information on adherence was available, it was mainly based on parental reports rather than objectively verified data. Therefore, the effect of occlusion therapy on long-term visual outcome could not be reliably analyzed. As adherence to amblyopia therapy is an important determinant of visual development after congenital cataract surgery [8], differences in treatment adherence may have influenced the observed visual outcomes and represent a potential confounding factor in our analysis. In addition, the severity and morphology of the cataract and the extent of preoperative visual deprivation could not be systematically quantified from the retrospective records. These factors may have influenced both timing of surgery and subsequent visual outcome and therefore represent potential confounding factors. Moreover, some patients with severe comorbidities had to be excluded from parts of the analysis due to unavailable visual acuity measurements. Furthermore, as this study was conducted at a tertiary referral center, referral patterns may have influenced the composition of our cohort. In particular, very young children requiring early cataract surgery may have been more likely to be treated at a university hospital, whereas older children may also have been treated at other institutions. Finally, the requirement of a minimum follow-up of six years may have led to selection bias, as patients lost to follow-up could not be included in the long-term outcome analysis, which might also have influenced the results.

## 5. Conclusions

Our findings support the prognostic importance of bilaterality in congenital cataract and indicate that good long-term visual outcomes can be achieved with different treatment options when appropriate postoperative care is provided. Furthermore, microphthalmia and prematurity were identified as important risk factors for additional surgical interventions after lensectomy. These findings may help to improve prognostic assessment and postoperative management in children with congenital cataract.

## Figures and Tables

**Table 1:** Epidemiological characteristics of the study population.

Parameter	Whole Cohort	Unilateral	Bilateral	*p*-Value
Number of eyes, n (%)	173	56 (32%)	117 (68%)	
Number of patients, n (%)	116	56 (48%)	60 (52%)	
Sex, n (%)				
Male	59 (51%)	24 (43%)	35 (58%)	0.14
Female	57 (49%)	32 (57%)	25 (42%)	
Age at surgery, median (IQR), months	3.9 (2.4–7.8)	4.9 (3.4–17.3)	3.4 (2.0–5.4)	0.0008
≤4 months, n (%)	90 (52%)	22 (39%)	58% (68)	0.02
≤2 months, n (%)	38 (42%)	7 (32%)	46% (31)	0.33
2–4 months, n (%)	52 (58%)	15 (68%)	54% (37)	
5–11 months, n (%)	53 (31%)	18 (32%)	30% (35)	0.86
≥12 months, n (%)	30 (17%)	16 (29%)	12% (14)	0.01
Refractive correction, n (%)				
Contact lens	117 (68%)	38 (68%)	68% (79)	>0.99
Spectacles	36 (21%)	7 (13%)	25% (29)	0.07
IOL	20 (12%)	11 (20%)	8% (9)	0.04

Data are presented for the total cohort. Age at surgery is shown as median and interquartile range (IQR). Data on sex are presented per patient, whereas age at surgery and refractive correction are presented per eye. *p*-values were calculated using the Mann–Whitney U test for age at surgery and Fisher’s exact test for categorical variables. Abbreviation: IOL, intraocular lens.

**Table 2:** Visual acuity after 6 years in unilateral and bilateral cases.

	n	Visual Acuity Mean ± SD (logMAR)	*p*-Value
Unilateral	56	1.0 ± 0.6	
Bilateral, all eyes	117	0.4 ± 0.3	<0.0001
Better eye	60	0.4 ± 0.3	<0.0001
Worse eye	57	0.5 ± 0.3	<0.0001

Comparison of best-corrected visual acuity (BCVA) six years after surgery between unilateral and bilateral congenital cataract. Data are presented per eye. In bilateral cases, better- and worse-seeing eyes were analyzed separately. Visual acuity is presented as mean BCVA in logMAR ± standard deviation (SD). *p*-values represent comparisons of VA between unilateral and bilateral cases and were calculated using unpaired *t*-tests.

**Table 3:** Impact of age at surgery on visual acuity in unilateral and bilateral congenital cataract after 6 years.

	Number of Eyes n (%)			Visual Acuity Mean ± SD (logMAR)			*p*-Value
	≤4 months	5–11 months	≥12 months	≤4 months	5–11 months	≥12 months	
Overall cohort	90 (52%)	53 (31%)	30 (17%)	0.7 ± 0.7	0.7 ± 0.6	0.6 ± 0.5	0.69
Unilateral	22 (39%)	18 (32%)	16 (29%)	1.3 ± 0.8	1.2 ± 0.8	0.9 ± 0.5	0.60
Bilateral (better eye)	33 (55%)	19 (32%)	8 (13%)	0.4 ± 0.4	0.4 ± 0.3	0.2 ± 0.2	0.23

Visual acuity in unilateral and bilateral (better eye) cataract according to age at surgery (≤4 months, 5–11 months, ≥12 months). Data are presented per eye. Visual acuity is presented as mean best-corrected visual acuity (BCVA) in logMAR ± standard deviation (SD). *p*-values represent comparisons of visual acuity between age groups and were performed using Kruskal–Wallis test.

**Table 4:** Impact of postoperative refractive correction on visual acuity after 6 years.

	Contact Lens	Spectacles	IOL	*p*-Value
Number of eyes, n (%)	117 (68%)	36 (21%)	20 (12%)	
Mean age at surgery, months	4.9 ± 6.3	7.5 ± 10.5	53.0 ± 41.7	<0.0001
BCVA (logMAR), mean ± SD	0.67 ± 0.61	0.79 ± 0.71	0.64 ± 0.51	0.67

Visual acuity and mean age at surgery according to the type of refractive correction. Data are presented per eye. Age at surgery and visual acuity are presented as mean ± standard deviation (SD). Visual acuity is shown as best-corrected visual acuity (BCVA) in logMAR. *p*-values represent comparisons between refractive correction groups and were calculated using the Kruskal–Wallis test. Abbreviation: IOL, intraocular lens.

**Table 5:** Impact of comorbidities on visual acuity after 6 years.

	Number of Eyes n (%)		Visual Acuity Mean ± SD (logMAR)		*p*-Value
	Comorbidity	No comorbidity	Comorbidity	No comorbidity	
Unilateral	22 (39%)	34 (61%)	1.1 ± 0.7	0.9 ± 0.6	0.30
Bilateral (better eye)	19 (32%)	41 (68%)	0.4 ± 0.2	0.3 ± 0.2	0.36

Comparison of visual acuity between eyes of patients with and without comorbidities. Comorbidities included microphthalmia, persistent fetal vasculature (PFV), prematurity, developmental retardation and syndromic diseases. Visual acuity is presented as mean best corrected visual acuity (BCVA) in logMAR ± standard deviation (SD). *p*-values represent comparisons of VA between patients with and without comorbidities and were calculated using unpaired *t*-tests.

**Table 6:** Association between comorbidities and the need for reoperation..

	No Reoperation n (%)	Reoperation n (%)	*p*-Value
No comorbidity	99 (87.6%)	14 (12.4%)	
Microphthalmia	7 (50%)	7 (50%)	0.0021
Persistent fetal vasculature	17 (77.3%)	5 (22.7%)	0.1969
Developmental delay	17 (73.9%)	6 (26.1%)	0.1083
Prematurity	5 (55.6%)	4 (44.4%)	0.0266
Other comorbidities	27 (75%)	9 (25%)	0.1086

Association between comorbidities and the need for additional surgical procedures. Reoperations included anterior chamber revision and secondary capsulotomy. Data are presented per eye. *p*-values were calculated using Fisher’s exact test.

**Table 7:** Logistic regression analysis of factors associated with low vision.

	Univariable Analysis		Multivariable Analysis	
	OR	95% CI	OR	95% CI
Unilateral cataract	5.56	2.72–11.36	6.3	2.9–13.7
Female sex	0.97	0.53–1.8	0.83	0.41–1.7
Age at surgery (continuous)	1.00	0.85–1.18	–	–
Age group at surgery (reference: ≥12 months)				
≤4 months	0.96	0.42–2.20	1.2	0.29–5.0
5–11 months	0.83	0.34–2.03	0.87	0.22–3.5
Presence of comorbidities	1.90	1.01–3.58	1.7	0.82–3.6
Revision surgery	1.40	0.64–3.05	1.4	0.56–3.5

Odds ratios (ORs) and 95% confidence intervals (CIs) of the univariable and multivariable logistic regression measuring the association of parameters with low vision by the definition of the World Health Organization (visual acuity > 0.5 logMAR, 20/70 Snellen or worse). Age group analyses were performed using patients 12 months of age or later (time of surgery) as a reference. Comorbidities included microphthalmia, persistent fetal vasculature, developmental retardation, prematurity and syndromic diseases. Revision surgery included anterior chamber revision and secondary capsulotomy. The multivariable model included all variables but age, which was included as a continuous variable. Abbreviations: 95% CI, 95% confidence interval.

## Data Availability

The original contributions presented in this study are included in the article. Further inquiries can be directed to the corresponding author.

## Abbreviations

The following abbreviations are used in this manuscript:

BCVA	Best-corrected visual acuity
logMAR	Logarithm of the minimum angle of resolution
IOL	Intraocular lens
SD	Standard deviation
VA	Visual acuity
PFV	Persistent fetal vasculature
OR	Odds ratio
CI	Confidence interval
TAPS	Toddler Aphakia and Pseudophakia Studies
IATS	Infant Aphakia Treatment Study
